# Automatic peak assignment and visualisation of copolymer mass spectrometry data using the ‘genetic algorithm’

**DOI:** 10.1002/rcm.8654

**Published:** 2020-02-12

**Authors:** James S. Town, Yuqui Gao, Ellis Hancox, Evelina Liarou, Ataulla Shegiwal, Christophe J. Atkins, David Haddleton

**Affiliations:** ^1^ Department of Chemistry University of Warwick Warwick, UK

## Abstract

Copolymer analysis is vitally important as the materials have a wide variety of applications due to their tunable properties. Processing mass spectrometry data for copolymer samples can be very complex due to the increase in the number of species when the polymer chains are formed by two or more monomeric units. In this paper, we describe the use of the genetic algorithm for automated peak assignment of copolymers synthesised by a variety of polymerisation methods. We find that in using this method we are able to easily assign copolymer spectra in a few minutes and visualise them into heat maps. These heat maps allow us to look qualitatively at the distribution of the chains, by showing how they alter with different polymerisation techniques, and by changing the initial copolymer composition. This methodology is simple to use and requires little user input, which makes it well suited for use by less expert users. The data outputted by the automatic assignment may also allow for more complex data processing in the future.

## 1 INTRODUCTION

Copolymers represent a wide range of materials encompassing different chemical, mechanical, and thermal properties, which can be somewhat tuned by altering the structure and compositional make‐up of the copolymer chain.^1^, ^2^, ^3^, ^4^, ^5^, ^6^ It is due to the relationship between synthetic methodology and tuneable properties that copolymers have found such diverse and essential applications.^7^, ^8^, ^9^, ^10^, ^11^ Copolymers of vinyl monomers may be synthesised by a wide variety of polymerisation methods, including catalytic chain transfer polymerisation (CCTP),^12^, ^13^, ^14^ atom transfer radical polymerisation (ATRP),^15^, ^16^, ^17^ ionic polymerisation,^18^, ^19^ reversible addition‐transfer chain‐transfer polymerisation (RAFT),^20^, ^21^, ^22^ and sulphur‐free RAFT.^23^, ^24^, ^25^, ^26^ These polymerisation methods can lead to different challenges in mass spectrometry, from examination of labile end groups^27^, ^28^, ^29^, ^30^ (e.g., ATRP and RAFT) to higher dispersities leading to a wide *m/z* range to be covered^31^, ^32^, ^33^, ^34^ (e.g., in CCTP). These challenges become even more complex, often to the point of becoming intractable and unsolvable, in the case of copolymers due to the enormous number of different molecular species present in a material. Therefore, improvements in copolymer analysis methods are of significant interest.

Polymers are mixtures of different molecular species that become increasingly more complex when two or more monomers are introduced into the system. This complexity can make characterisation extremely difficult, especially during exact determination of the types of species that exist and their relative quantity. Previous approaches have included 2D nuclear magnetic resonance (NMR) methodology,^35^, ^36^, ^37^ liquid chromatography/mass spectrometry (LC/MS),^38^, ^39^ and 2D chromatography.^40^, ^41^, ^42^ All these approaches are complex and may not always give the detailed understanding of these copolymer materials that is required.

Data processing techniques for mass spectrometry data have become more of a relevant field as the complexity of data has increased, with more powerful mass spectrometers being used, and the technique has gained a wider use in industrial fields. Polymeromics is no exception to this; however, as with many aspects of mass spectrometry, this subfield lags behind the related fields (proteomics, petroleomics, lipidomics, etc.).

Kendrick mass defect (KMD) plots have proved invaluable to other areas of mass spectrometry, such as proteomics,^43^, ^44^ and have been applied rigorously to polymers. This includes improvements such as fractional base units^45^, ^46^ and slicing,^47^ which have, or are likely to in the near future, greatly improved their application to copolymer analysis.^48^ The benefit of using KMD plots is that they are applied to all the peaks in the sample, displaying all structures that have the base unit as horizontal lines. The downside, however, is that although these plots do simplify assignment by processing peaks into lines with the same KMD, the assignment must still be carried out manually, or by separate automation.

Mass remainder analysis (MaRA) as proposed by Nagy et al^49^ is similar to the Kendrick mass defect; however. it is much more simplified, utilising the division of a peak by one of the repeat units to allow for the separation of species into visual horizontal rows. Therefore, although it does give visualisation of the copolymer, it still requires manual assignment, and so this technique is very similar to Kendrick mass defect analysis^50^ and therefore shares similar drawbacks.

Willemse et al utilised matrix‐assisted laser desorption/ionisation (MALDI) data to develop contour plots for copolymer fingerprinting, using an array‐based assignment. They were able to track the progress of a polymerisation of a block copolymer and demonstrate the contour plot changing through their spectra.^51^ They, however, ran into issues with multiple assignments for a single peak, an issue that arose from the lower‐resolution mass spectrometers and the array methodology used due to the lower computational power available at the time of the study.

The genetic algorithm has been applied to mass spectrometry analysis of metabolite systems before. This methodology is used for predicting markers involved in the diagnosis of cancer in patients, alongside more traditional principal component analysis.^52^ Its purpose is different from the direct peak assignment that we will report in this work.

Genetic algorithm analysis has also been applied to tandem mass spectrometry data of glycosaminoglycans. This analysis, which is fast and accurate, allows for high throughput structural determination of these species by reducing the R groups to a binary sequence; however, such a binary sequence may be more difficult to implement on synthetic polymer samples due to the presence of different monomers.^53^

In this current work, we use the genetic algorithm to automatically assign peaks in MALDI‐TOF data for copolymer samples. As an example of the usefulness of the generated output, the data are used to generate simple visualisations of complex copolymer spectra, which will allow non‐expert users to analyse copolymer samples. We believe that the genetic algorithm peak assignment could lead to more advanced, automated copolymer analyses in the future.

## 2 METHODS

### 2.1 Matrix‐assisted laser desorption/ionisation time‐of‐flight mass spectrometry

MALDI‐TOF experiments were carried out using a Bruker (Bremen, Germany) Autoflex time‐of‐flight mass spectrometer, equipped with a 337 nm N_2_ laser, operating at 21 kV acceleration voltage in reflectron positive mode. Samples were prepared in tetrahydofuran (THF) (at 10 mg mL^−1^) with sodium iodide salt (1 mg mL^−1^) and a DCTB matrix (40 mg mL^−1^), with the only exception being the styrene‐methyl methacrylate copolymer which was prepared with silver trifluoroacetate (1 mg mL^−1^) as well as the sodium iodide salt. The solution was then spotted onto an MTP 382 ground steel target plate (Bruker) for analysis.

### 2.2 Mathematics and scripting

**Figure 1:**
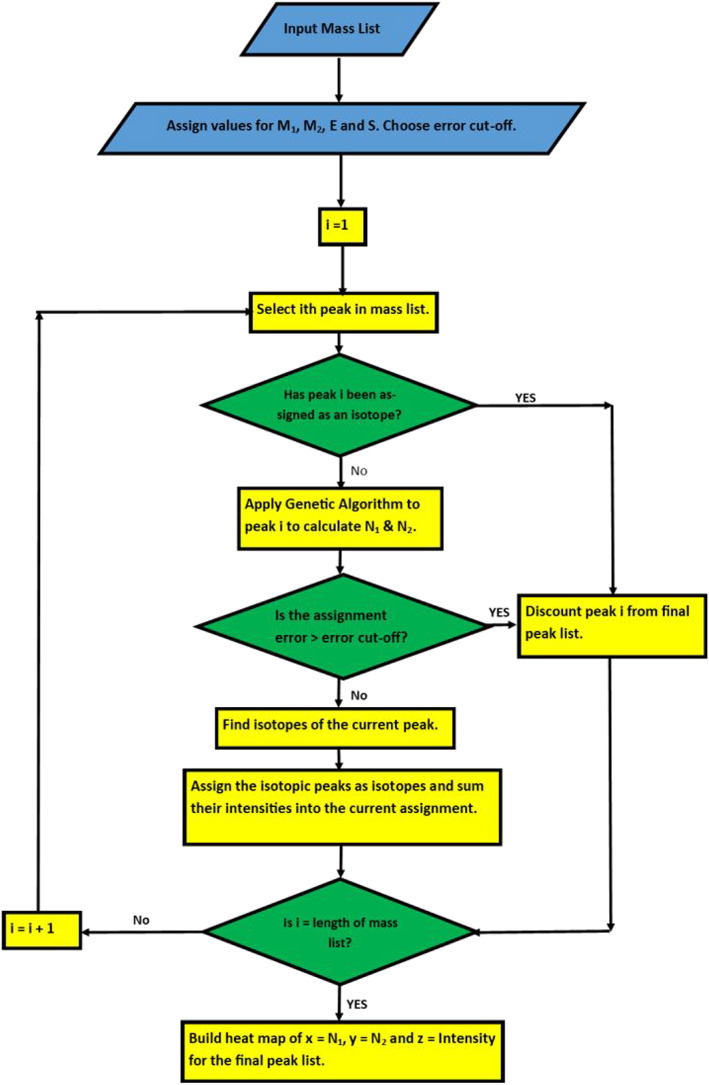
Flow chart of the Matlab script utilised in this work

Matlab was utilised to script all the data analysis, including the production of the graphs shown throughout this article (Figure 1). To generate automated peak picking we utilised the genetic algorithm function found in the global optimisation toolbox as it allowed for integer constraints; the parameters to provide the fastest, correct assignment are described in the supporting information. Equation 2 shows the mass values of end groups (*E*), monomer 1 (*M*_1_), monomer 2 (*M*_2_) and ionising salt (*S*) which are all known given a single manually assigned peak. The genetic algorithm, therefore, is utilised to find the minimum value of error by adjusting the number of monomer 1 and monomer 2 units (*N*_1_ and *N*_2_):

(1) Error=theoretical mass−experimental mass

(2) $$\text{Error}=E+N_{1}\times M_{1}+N_{2}\times M_{2}+S-\text{experimental mass}$$

In a perfectly calibrated mass spectrum we would be able to minimise this equation to 0. However, no mass spectrometry is ever perfect, and therefore there will always be an associated error. The script, therefore, includes an adjustable error cut‐off which, after it has finished assigning all peaks, is then used to remove any assignments not satisfying this error. We recommend an error cut‐off of 0.1 *m/z* units or below, as this is perfectly achievable even with external calibration in relatively low‐resolution TOF instruments.

Once a peak has been assigned, to allow for better representation of the intensity in the mass spectra, the script attempts to find all the isotopic peaks which relate to the assigned peak and sum up their intensities. This is to avoid higher‐molecular‐weight peaks being underrepresented by the intensity of their monoisotopic mass, which is used to calculate the assignment, as this is not the highest intensity peak for carbon‐based polymers with molecular masses above ~2000 Daltons depending on the chemical formula. This is achieved by attempting to find a peak, which is both 1 *m/z* unit higher, within the assignment error, and has an intensity which is less than a selected multiple of that of the original peak. This intensity factor is not set to allow for adjustment for samples with halides, or other elements with more complex isotopic distributions. Peaks which are determined to be isotopes of a previous peak are discounted from being assigned later, and are therefore not put through the genetic algorithm. The genetic algorithm is by far the most computationally expensive part of the code; therefore, discounting these peaks before assignment allows for less processing time.

## 3 RESULTS

### 3.1 Optimisation of genetic algorithm parameters

The parameters used in the genetic algorithm were optimised using a poly(methyl acrylate‐ethyl acrylate) statistical copolymer, with a 50/50 ratio between the two monomers, synthesised by Cu(0)‐mediated SET‐LRP. The optimisations carried out are presented in the supporting information, including the specifications for the laptop used to carry out the Matlab script, and the final parameters utilised for the algorithm, which are in the following table:

**Table:** 

Initial population	Elite children	Function tolerance	Max generations
Permeations/4	0.7 * previous population	10^100^	40

Permeations is a value calculated as the number of all possible combinations of the two monomers calculated as follows:

(3) $${\mathrm{DP}}_{\text{pred}}=\frac{\frac{m}{z}\text{maximum}}{M_{\text{maximum}}}$$

(4) $$\text{Permeations}=\frac{{\mathrm{DP}}_{\text{pred}}!}{\left(\text{DPpred}-N\left(\text{monomers}\right)\right)!}$$

where *DP*_*pred*_ represents the predicted maximum degree of polymerisation that the chains can take, calculated by dividing the maximum *m/z* value in the dataset by the mass of the highest‐mass monomer being used for assignment. The Permeations value is therefore calculated using the predicted maximum degree of polymerisation (*DP*_*pred*_) and the number of different monomers (*N* [*monomers*]). By altering the initial population based on the number of possible results, the algorithm does not have to be manually altered for polymers with more or fewer possibilities, giving accurate results without user input.

With the current optimisation of this algorithm, the Matlab script currently takes 17 seconds on a > 900 peak dataset, reducing it to 110 species with good repeatability.

### 3.2 MA/EA statistical copolymers

**Figure 2:**
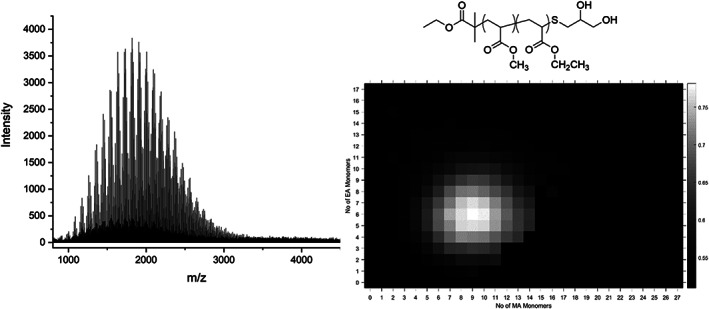
Methyl acrylate‐co‐ethyl acrylate 50/50 mol% synthesised by photomediated SET‐LRP MALDI‐TOF‐MS (left), structure of methyl acrylate‐co‐ethyl acrylate with a substituted thiol end group (top right), and heat map produced from the data (bottom right)

**Figure 3:**
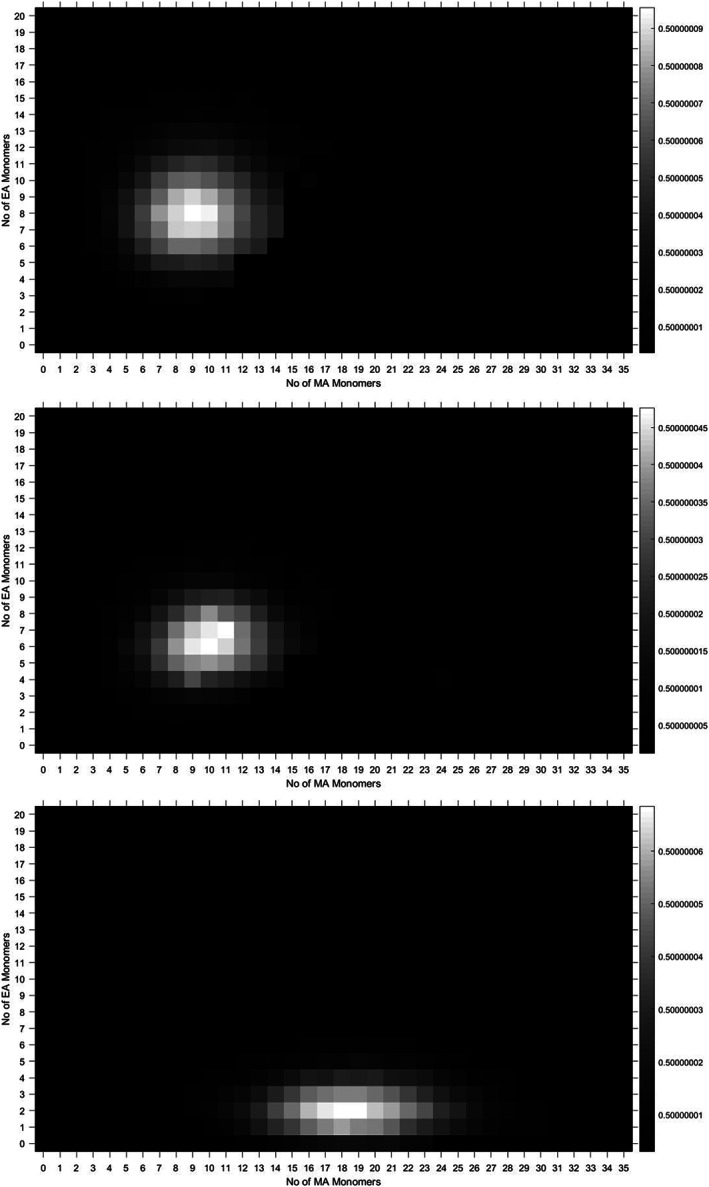
Heat maps visualising the data for methyl‐acrylate‐co‐ethyl acrylate statistical copolymers with ratios of 50/50 (top), 70/30 (middle), and 90/10 (bottom)

The automatic peak assignment by the genetic algorithm is used to generate a heat map with *N*_1_ on the *x*‐axis, *N*_2_ on the y‐axis and a transformed intensity at the colour gradient. This provides visualisation of the copolymers, allowing for simple qualitative comparison. The heat maps for methyl acrylate‐co‐ethyl acrylate with monomer ratios of 50/50, 60/40, 70/30, 80/20 and 90/10 (Table S1, supporting information) are distinct in their overall shape as the more MA than EA in the monomer ratio , the shallower the gradient the heat map appears to have (Figure 2). The heat maps also provide a visual diagnostic for the assignment if the distribution provided on the heat map has no gaps, which could imply peaks missed by the algorithm, or higher intensity points lying outside the main distribution, which would imply peaks which were assigned incorrectly. The 70/30 copolymer displays this, as its width is due to a misassigned peak on the very far right of the heat map (Figure 3).

**Figure 4:**
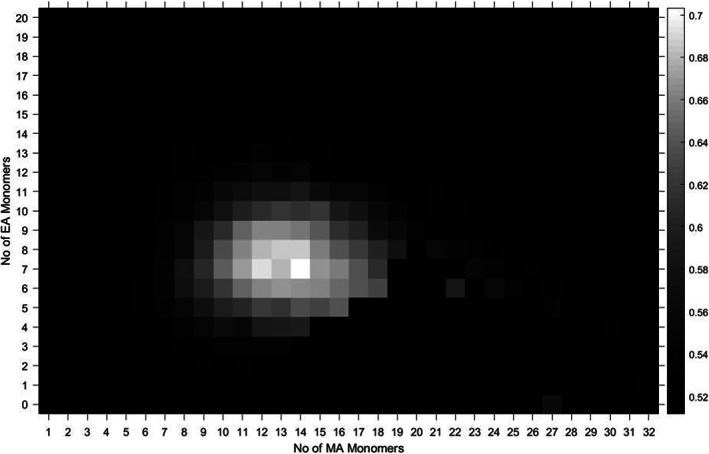
60/40 methyl acrylate‐co‐ethyl acrylate statistical copolymer synthesised by photomediated SET‐LRP, showing the issue of misassignment of isotopic peaks

It is therefore possible to use the heat map to find the peaks which have been missed or which have been assigned incorrectly in the original spectra. This allows for the visualisation as a diagnostic tool for the genetic algorithm assignment. The assignment is reliant on good calibration, as this will minimise the error that is set as a cut‐off for correct assignments. The example MA/EA 60/40 heat map, in Figure 4, shows this effect of poor calibration. In this case it would appear that several isotopic peaks have been assigned as real species. This indicates that the calibration led to them not being correctly assigned as isotopes, and therefore they were not removed from the potential assignments. Falsely assigned isotopic peaks also have the downside of causing the relative intensities in the heat map, and the absolute intensities in the genetic algorithm output, to be less representative of the real data, as the isotopic distribution is not correctly summed into the real assigned peak.

**Figure 5:**
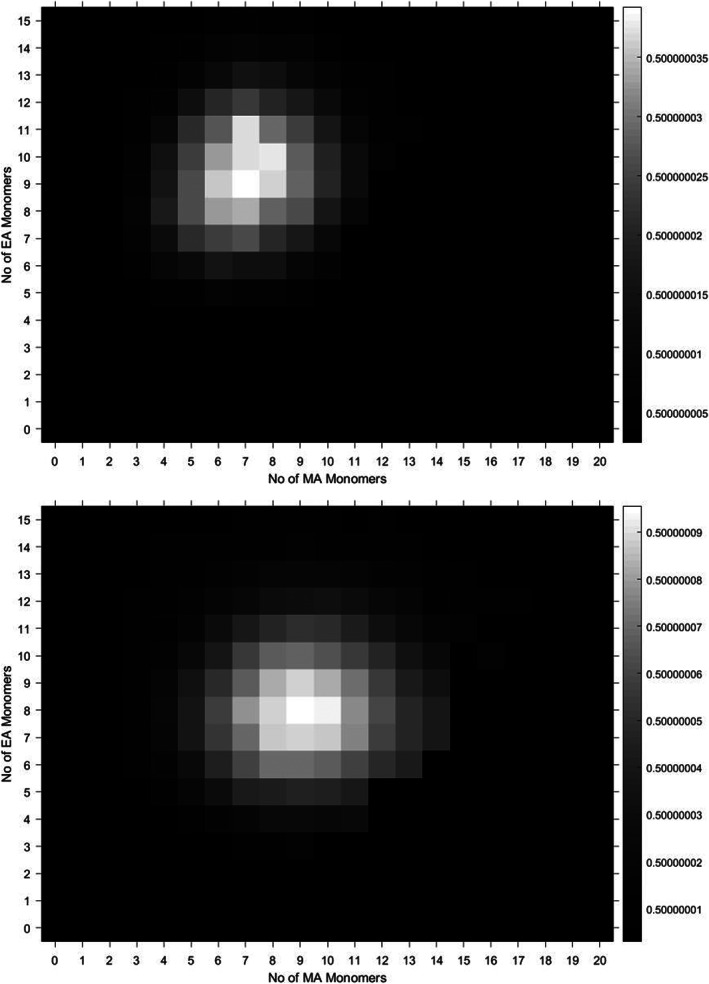
50/50 methyl acrylate‐co‐ethyl acrylate statistical copolymer synthesised by copper(0) wire (top) and photomediated Cu(II) (bottom) SET‐LRP

Comparing methyl acrylate‐co‐ethyl acrylate copolymers made by two different synthetic chemists using two different and distinct forms of copper‐mediated living radical polymerisation (one photomediated,^54^, ^55^, ^56^ the other using a copper(0) wire system^57^, ^58^, ^59^, ^60^), we can draw some simple conclusions about the synthesis qualitatively (Figure 5). By examining the heat maps of a copolymer with a 50/50 mole% composition side by side, we can see that the copper wire system was more controlled, in that the distribution of the copolymer spectra seems to be less dispersed. This form of examination is a new way of looking at synthetic copolymerisations, as we can examine an under‐evaluated area of synthetic control, the control over the composition of the chains.

### 3.3 Analysis of MMA/EMA diblock copolymers

**Figure 6:**
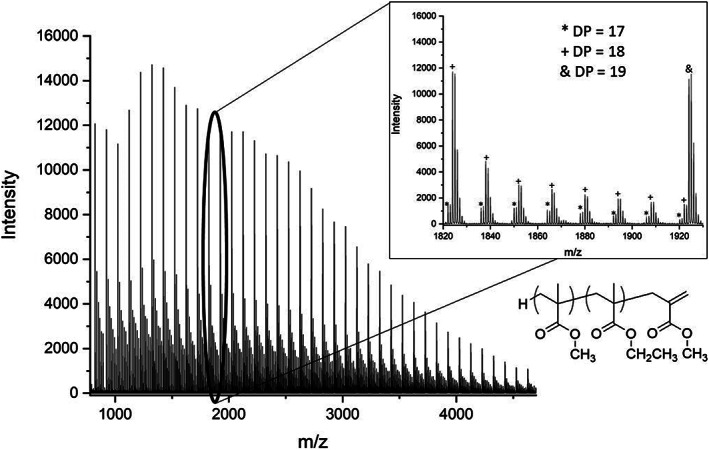
Methyl methacrylate‐co‐ethyl methacrylate diblock full spectrum; the part that is zoomed shows the overlapping of isotopic distributions between different species

When a 10 MMA 10 EMA diblock, synthesised using a combination of CCTP^12^, ^13^ and sulphur‐free RAFT (SF RAFT),^23^, ^24^, ^25^ was analysed by MALDI‐TOF, its spectrum had interesting features as it did not contain a normal compositional distribution with narrow dispersity (Figure 6). The spectrum was then analysed with the genetic algorithm and displayed as a heat map. One of the issues in this spectrum is how broad it is; it is found that in spectra over this range of masses it is difficult to get a very high accuracy of calibration. Therefore, the assignment error is higher in some of the real peaks, which, when accounted for, leads to some misassignments. Lower‐abundance species have overlapping isotopic distributions with other higher abundance species, and therefore some assignments are also lost when the intensity cut‐off factor of our isotopic distribution assignment is too high. This is because peaks which come after the lower‐abundance species can be assigned as isotopes of those lower‐abundance peaks, similar to the MA/EA system.

**Figure 7:**
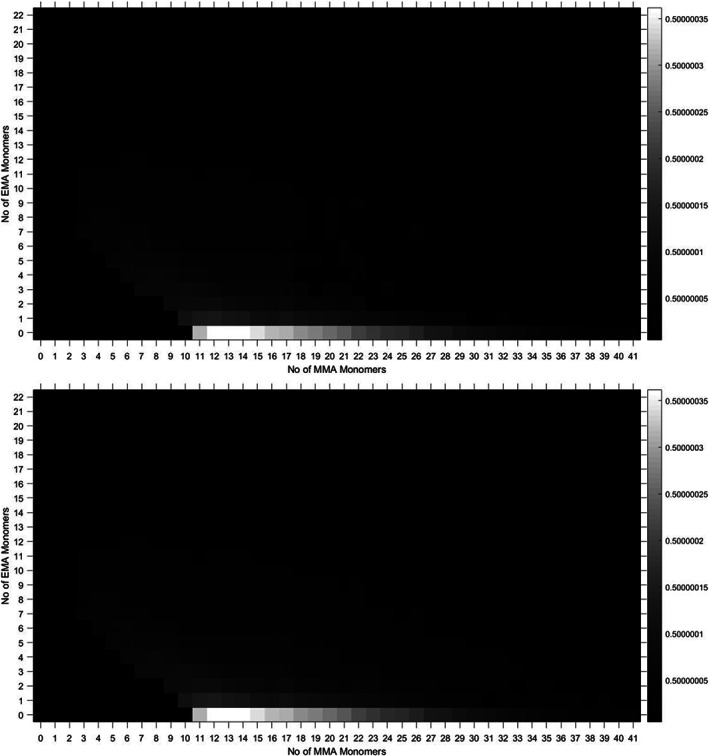
Methyl methacrylate‐co‐ethyl methacrylate diblock, synthesised by catalytic chain transfer polymerisation and sulphur‐free reversible addition‐transfer chain‐transfer polymerisation. Isotopic intensity issue shown (top), and then resolved (bottom)

The heat map in Figure 7 shows that the sample contains high amounts of PMMA homopolymer. This implies that the incorporation of the macromonomer into a block copolymer was incomplete, even though the monomer conversion was taken to a high percentage (>95%). The polymer has a broad dispersity, around 1.7, which could mean that higher‐molecular‐weight chains contain more of the EMA than the MMA polymers; however, the limitations of the mass spectrometer prevent the accurate analysis of copolymer distributions having molecular weight >10 000. The other significant difference between this and the previous example is the greatly increased number of molecular species. This is because the number of copolymer species observed in a diblock copolymer sample is related to the molecular weight distribution of the second block of the diblock, which is likely also to be very broad.

This displays the importance of mass spectrometry relative to bulk measurements which are traditionally used in polymer characterisation, such as 1D NMR, which would not be able to show this homopolymer problem; instead it would provide an average monomer incorporation in all polymeric chains. Using MALDI‐TOF‐MS, in collaboration with the genetic algorithm peak assignment, we are able to display the data with ease.

### 3.4 Analysis of an MMA‐styrene statistical copolymer

**Figure 8:**
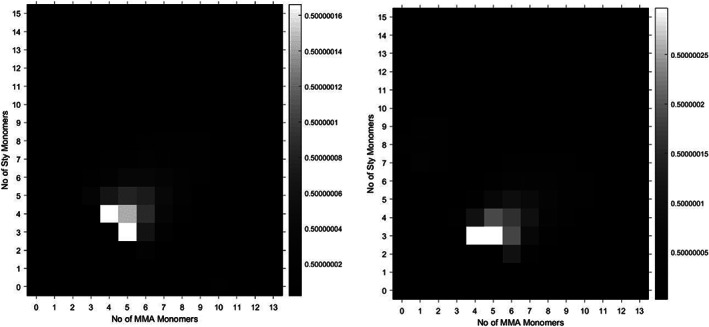
Styrene‐co‐methyl methacrylate statistical copolymer synthesised by bulk free radicals, using AgTFA (left) and NaI (right) as a cationising agent

A methyl methacrylate‐styrene statistical copolymer synthesised by free radicals in bulk (supporting information) demonstrates some of the effect of reactivity ratios on the number of observed species in the mass spectrum (Figure 8). The reactivity ratios of MMA‐styrene copolymers have been shown to be *r*_MMA_ = 0.51 and *r*_Styrene_ = 0.49^61^; this implies that the reaction tends toward a slightly alternating sequence. This, therefore, would lead to a reduction in the number of species, as alternating polymers would have a maximum of three species per degree of polymerisation. Using the genetic algorithm assignment to build a visual heat map, we can observe that the width of the distribution appears very thin. This occurs regardless of whether we use sodium or silver salt, showing that this is not merely an effect of ionisation efficiency, as methacrylate and styrene species ionise more efficiently with different cation species.

## 4 CONCLUSIONS

The genetic algorithm has been used for the automated assignment of copolymer mass spectra, with high accuracy and efficiency. Its utilisation on presenting usually complex mass spectra as simple heat maps allows for the qualitative comparison of data, in the case of low‐molecular‐weight copolymers. Improvements are still to be made on the implementation of the data processing methodology, such as the way in which isotopic distributions are handled means that the methodology is probably ignoring certain overlapping species. To overcome this would require either higher‐resolution instrumentation or predicting the amount of intensity within a certain overlapping peak which is to be allocated to each constituent species. Other ways to alter the approach discussed here would be to allow for the assignment of multiple end groups, as our approach only assigns all copolymer peaks with a given end group. This is simple to overcome in the genetic algorithm methodology; however, it will greatly increase the computational power required to run such a script. The output of having all copolymer peaks assigned, in a simple and automatic manner, allows for a future of more advanced analysis of very complex datasets.

## Supporting information


**Table S1**. Methyl acrylate and ethyl acrylate monomer volumes for 20 degree of polymerisation polymers.
