# Supplementary material for: Automatic peak assignment and visualisation of copolymer mass spectrometry data using the ‘genetic algorithm’
Source: Rapid Commun Mass Spectrom. 2020 Feb 12;34(Suppl 2):e8654. doi: 10.1002/rcm.8654 (PMC7507196; doi:10.1002/rcm.8654)
Supplement: Supplementary file 1 — Table S1. Methyl acrylate and ethyl acrylate monomer volumes for 20 degree of polymerisation polymers. [file RCM-34-e8654-s001.docx]

**Automatic Peak Assignment and Visualisation of Copolymer Mass Spectrometry Data Using the Genetic Algorithm – Supporting Information**

**Optimization of Parameters for the Genetic Algorithm Code**

Computational parameters for the code were determined by running optimizations on a poly (methyl acrylate – ethyl acrylate) 50/50 copolymer synthesised by Cu (0) mediated SET LRP. The optimizations were performed on a HP Elitebook, with an Intel® Core™ i5-6200U CPU 2.30GHz processor and 16 GB of RAM. Number of peaks assigned includes the assigned isotopic peaks, which seems to have a cap of 110. The number of peaks put into the code was 992 which includes many noise peaks, showing the algorithms power of exact determination. The qualitative assessment is simply looking at the assignment and noting any gaps in the distribution. This is because a missing peak can be due to the absence of an actual assignment, leading to a gap, or the absence of an isotopic peak, which is harder to determine by eye.

| Parameter set | Initial Population Size | Elite Count (as fraction of population size | Function Tolerance | Max Generations | Time taken (seconds) | Number of assigned peaks | Ending Condition for Assignment | Qualitative assessment of assignment |
| --- | --- | --- | --- | --- | --- | --- | --- | --- |
| 1 | 248 | 0.5 | 10^-1000^ | 75 | 56 | 109 | Function Tolerance | Very good |
| 2 | 331 | 0.5 | 10^-1000^ | 75 | 67 | 110 | Function Tolerance | Very good |
| 3 | 496 | 0.5 | 10^-1000^ | 75 | 62 | 110 | Function Tolerance | Very good |
| 4 | 992 | 0.5 | 10^-1000^ | 75 | 129 | 110 | Function Tolerance | Very good |
| 5 | 198 | 0.5 | 10^-1000^ | 75 | 30 | 108 | Function Tolerance | One obvious missed assignment |
| 6 | 124 | 0.5 | 10^-1000^ | 75 | 36 | 102 | Function Tolerance | Several obvious missed assignments |
| 7 | 99 | 0.5 | 10^-1000^ | 75 | 21 | 101 | Function Tolerance | Several obvious missed assignments |
| 8 | 50 | 0.5 | 10^-1000^ | 75 | 25 | 84 | Function Tolerance | Very incomplete assignment |

|  | Number of Assigned Peaks | Time Taken (seconds) | Qualitative Assessment of the Assignment |
| --- | --- | --- | --- |
| Parameter Set 1 Repeat 1 | 109 | 56 | Very Good |
| Parameter Set 1 Repeat 2 | 110 | 52 | Very Good |
| Parameter Set 1 Repeat 3 | 108 | 55 | One Missed Assignment |
| Parameter Set 1 Repeat 4 | 110 | 51 | Very Good |
| Parameter Set 1 Repeat 5 | 110 | 48 | Very Good |

| Parameter set | Initial Population Size | Elite Count (as fraction of population size) | Function Tolerance | Max Generations | Time taken (seconds) | Number of assigned peaks | Ending Condition for Assignment | Qualitative assessment of assignment |
| --- | --- | --- | --- | --- | --- | --- | --- | --- |
| 9 | 248 | 0.1 | 10^-1000^ | 75 | 86 | 109 | Function Tolerance | Very Good |
| 10 | 248 | 0.2 | 10^-1000^ | 75 | 74 | 110 | Function Tolerance | Very Good |
| 11 | 248 | 0.3 | 10^-1000^ | 75 | 71 | 109 | Function Tolerance | Very Good |
| 12 | 248 | 0.4 | 10^-1000^ | 75 | 67 | 109 | Function Tolerance | Very Good |
| 13 | 248 | 0.5 | 10^-1000^ | 75 | 56 | 109 | Function Tolerance | Very Good |
| 14 | 248 | 0.6 | 10^-1000^ | 75 | 49 | 108 | Function Tolerance | Missing 1 assignment |
| 15 | 248 | 0.7 | 10^-1000^ | 75 | 29 | 110 | Function Tolerance | Very Good |
| 16 | 248 | 0.8 | 10^-1000^ | 75 | 31 | 109 | Function Tolerance | Very Good |
| 17 | 248 | 0.9 | 10^-1000^ | 75 | 24 | 108 | Function Tolerance | Missing 1 assignment |
| 18 | 248 | 0.99 | 10^-1000^ | 75 | 24 | 57 | Function Tolerance | Incomplete Assignment |

|  | Number of Assigned Peaks | Time Taken (seconds) | Qualitative Assessment of the Assignment |
| --- | --- | --- | --- |
| Parameter Set 17 Repeat 1 | 109 | 26 | Missing 1 assignment |
| Parameter Set 17 Repeat 2 | 109 | 25 | Missing 1 assignment |
| Parameter Set 17 Repeat 3 | 108 | 26 | Missing 2 assignments |
| Parameter Set 17 Repeat 4 | 108 | 26 | Missing 2 assignments |
| Parameter Set 17 Repeat 5 | 110 | 24 | Very Good |

|  | Number of Assigned Peaks | Time Taken (seconds) | Qualitative Assessment of the Assignment |
| --- | --- | --- | --- |
| Parameter Set 15 Repeat 1 | 110 | 38 | Very Good |
| Parameter Set 15 Repeat 2 | 110 | 38 | Very Good |
| Parameter Set 15 Repeat 3 | 110 | 38 | Very Good |
| Parameter Set 15 Repeat 4 | 109 | 38 | 1 Missing Assignment |
| Parameter Set 15 Repeat 5 | 110 | 38 | Very Good |

| Parameter set | Initial Population Size | Elite Count (as fraction of population size) | Function Tolerance | Max Generations | Time taken (seconds) | Number of assigned peaks | Ending Condition for Assignment | Qualitative assessment of assignment |
| --- | --- | --- | --- | --- | --- | --- | --- | --- |
| 19 | 248 | 0.7 | 10^-1000^ | 75 | 38 | 110 | Function Tolerance | Very Good |
| 20 | 248 | 0.7 | 10^-100^ | 75 | 39 | 109 | Function Tolerance | Very Good |
| 21 | 248 | 0.7 | 10^-10^ | 75 | 38 | 110 | Function Tolerance | Very Good |
| 22 | 248 | 0.7 | 10^-1^ | 75 | 41 | 110 | Function Tolerance | Very Good |
| 23 | 248 | 0.7 | 10^0^ | 75 | 36 | 110 | Function Tolerance | Very Good |
| 24 | 248 | 0.7 | 10^1^ | 75 | 36 | 110 | Function Tolerance | Very Good |
| 25 | 248 | 0.7 | 10^10^ | 75 | 39 | 108 | Function Tolerance | One Missing Assignment |
| 26 | 248 | 0.7 | 10^100^ | 75 | 36 | 110 | Function Tolerance | Very Good |
| 27 | 248 | 0.7 | 10^1000^ | 75 | 8 | 35 | Function Tolerance | Incomplete assignment |
| 28 | 248 | 0.7 | 10^308.25471555991^ | 75 | 36 | 110 | Function Tolerance | Very Good |

|  | Number of Assigned Peaks | Time Taken (seconds) | Qualitative Assessment of the Assignment |
| --- | --- | --- | --- |
| Parameter Set 26 Repeat 1 | 109 | 36 | Very Good |
| Parameter Set 26 Repeat 2 | 109 | 36 | Very Good |
| Parameter Set 26 Repeat 3 | 109 | 36 | Very Good |
| Parameter Set 26 Repeat 4 | 110 | 36 | Very Good |
| Parameter Set 26 Repeat 5 | 110 | 35 | Very Good |

| Parameter set | Initial Population Size | Elite Count (as fraction of population size) | Function Tolerance | Max Generations | Time taken (seconds) | Number of assigned peaks | Ending Condition for Assignment | Qualitative assessment of assignment |
| --- | --- | --- | --- | --- | --- | --- | --- | --- |
| 29 | 248 | 0.7 | 10^100^ | 75 | 36 | 109 | Function Tolerance | Very Good |
| 30 | 248 | 0.7 | 10^100^ | 50 | 23 | 110 | Maximum Generations | Very Good |
| 31 | 248 | 0.7 | 10^100^ | 25 | 12 | 108 | Maximum Generations | One missing assignment |
| 32 | 248 | 0.7 | 10^100^ | 5 | 6 | 81 | Maximum Generations | Incomplete Assignment |
| 33 | 248 | 0.7 | 10^100^ | 35 | 16 | 109 | Maximum Generations | Two missing Assignments |
| 34 | 248 | 0.7 | 10^100^ | 45 | 21 | 110 | Maximum Generations | Very Good |
| 35 | 248 | 0.7 | 10^100^ | 40 | 19 | 110 | Maximum Generations | Very Good |
| 36 | 248 | 0.7 | 10^100^ | 65 | 24 | 110 | Function Tolerance | Very Good |
| 37 | 248 | 0.7 | 10^100^ | 60 | 21 | 109 | Function Tolerance | One Missing Assignment |
| 38 | 248 | 0.7 | 10^100^ | 52 | 22 | 109 | Function Tolerance | Very Good |

|  | Number of Assigned Peaks | Time Taken (seconds) | Qualitative Assessment of the Assignment |
| --- | --- | --- | --- |
| Parameter Set 38 Repeat 1 | 109 | 23 | Very Good |
| Parameter Set 38 Repeat 2 | 110 | 24 | Very Good |
| Parameter Set 38 Repeat 3 | 110 | 21 | 3 Missing Peaks |
| Parameter Set 38 Repeat 4 | 109 | 22 | Very Good |
| Parameter Set 38 Repeat 5 | 110 | 23 | Very Good |

|  | Number of Assigned Peaks | Time Taken (seconds) | Qualitative Assessment of the Assignment |
| --- | --- | --- | --- |
| Parameter Set 30 Repeat 1 | 110 | 22 | Very Good |
| Parameter Set 30 Repeat 2 | 110 | 21 | Very Good |
| Parameter Set 30 Repeat 3 | 110 | 21 | Very Good |
| Parameter Set 30 Repeat 4 | 109 | 20 | Very Good |
| Parameter Set 30 Repeat 5 | 109 | 21 | One Peak Missing |

|  | Number of Assigned Peaks | Time Taken (seconds) | Qualitative Assessment of the Assignment |
| --- | --- | --- | --- |
| Parameter Set 35 Repeat 1 | 110 | 17 | Very Good |
| Parameter Set 35 Repeat 2 | 110 | 17 | Very Good |
| Parameter Set 35 Repeat 3 | 110 | 17 | Very Good |
| Parameter Set 35 Repeat 4 | 110 | 17 | Very Good |
| Parameter Set 35 Repeat 5 | 110 | 16 | Very Good |

**Synthetic Procedures**

***General procedure for photo-induced polymerization*** *- example target PMA-*s*-EA*

CuBr_2_, Me_6_Tren, EBiB and total volume were kept constant and volumes of MA/EA were varied to achieve polymers of the same length (DP 20) but different monomer distributions. These values were taken from the quantities for a EBiB initiated, DP 20 poly(methyl acrylate) of 8.8 ml total volume. CuBr_2_ (10.9 mg, 0.02 eq.) was dissolved in DMSO (4.4 ml) by sonication, followed by addition of Me_6_Tren (78 µl, 0.12 eq.). MA and EA (volumes given in Table X) were added according to the desired monomer distributions. The mixture was degassed with nitrogen for 10 min before adding EBiB initiator (359 µl, 1 eq.) and further degassed for 5 min. The reaction was then left under an ultraviolet lamp overnight. The resulting polymer was dissolved in minimum acetone and precipitated in 50:50 deionised H_2_O:MeOH. Product was dissolved in acetone and passed through neutral alumina, solvent removed and dried in a vacuum oven at 25 °C overnight.

**Table S1**. Methyl acrylate and ethyl acrylate monomer volumes for 20 degree of polymerisation polymers.

| **Entry** | **MA/EA ratio (%)** | **Volume MA (ml)** | **Volume EA (ml)** |
| --- | --- | --- | --- |
| A | 50/50 | 2.0 | 2.4 |
| B | 60/40 | 2.45 | 1.95 |
| C | 70/30 | 2.90 | 1.50 |
| D | 80/20 | 3.40 | 1.0 |
| E | 90/10 | 3.90 | 0.50 |

**Process for the synthesis of macromonomer (PMMA) by CCTP in emulsion.**

In a typical CCTP emulsion polymerisation, CoBF (0.096 g, 0.2222 mmol) was placed in a 250 mL round bottom flask together with a stirring bar. Nitrogen was purged in the flask for at least 1h. Subsequently, MMA (120 mL, 112.32 g, 1121.85 mmol) previously degassed for 30 min was added to the flask via a degassed syringe. The mixture was vigorously stirred under inert atmosphere until total dissolution of the catalyst. Meanwhile, ACVA (2.2 g, 7.888 mmol), SDS (1.8 g, 6.242 mmol) and 250 mL of water were charged into a three-neck, 500mL double jacketed reactor, equipped with a RTD temperature probe and an overhead stirrer. The mixture was purged with nitrogen and stirred at 325 rpm for at least 30 min. Subsequently, the mixture was heated under inert atmosphere. When the temperature in the reactor reached 70 °C, the addition of the MMA -CoBF solution started using a degassed syringe and a syringe pump (feeding rate= 2 mL/min, feeding time=60 min). When the addition was over, stirring continued for another 60 min under the same conditions. The number average molecular weight of the macromonomer was calculated by analysing the 1H NMR spectra.

**Process for the chain extension of macromonomer Poly(MMA) with EMA (DPn = 10) by Free-Radical polymerisation in emulsion.**

50 mL of PMMA latex (0.2411 g/mL) were diluted by adding 40 mL of water to achieve a 22.4% solid content. The resulting latex was charged in the reactor and purged with nitrogen for 30 min under stirring. Subsequently, the emulsion was heated. When the temperature in the reactor reached 80-82 °C and was stabilised, the simultaneous addition of EMA (13.64 mL, 12.509 g, 0.110 mol) and potassium persulfate aqueous solution (68.2 mg potassium persulfate in 13.64 mL of water), both previously degassed for 30 min started by the use of degassed syringes and a syringe pump (feeding rate=0.16 mL/min, feeding time = 232 min). When the addition was over, stirring continued for another 60 min under the same conditions.

**Typical procedure for the synthesis of the statistical P(MA_x_-*sta*t-EA_y_) copolymers *via* Cu(0) wire mediated Reversible Deactivation Radical Polymerization.**

A glass vial was charged with Me_6_Tren (0.18 eq.), Cu(ΙΙ)Br_2_ (0.05 eq.) and DMSO (4 mL). MA (x eq.), EA (y eq.), EBiB (1 eq.) and pre-activated copper wire (5 cm) wrapped around a stirring bar were added to the complex solution and the vial was septum sealed. N_2_ sparging was applied to the solution for 15 min for the removal of oxygen, and the polymerization was left to commence at ambient temperature. Once quantitative conversion was verified through ^1^H NMR analysis, a sample was taken and passed through a short column of neutral alumina for the removal of dissolved copper salts prior to SEC analysis in THF.

**Typical procedure for thiobromine substitution.**

The obtained polymers were purified through precipitation in H_2_O-MeOH solutions (70-30 % *v/v*) and dried under vacuum. Subsequently, in the purified P(MA_x_-*stat*-EA_y_) (1 mol equiv.) copolymers, 1-thio glycerol (1.5 eq.), triethylamine (1.5 eq.), acetone (2 mL) and a stirrer bar were added and the reaction was left to commence for 2 hours. The thioglycerol functionalized copolymers were then used for mass spectroscopy analysis.

**Poly (methyl methacrylate – co – Styrene)**

A statistical copolymer of methyl methacrylate and styrene was prepared by introducing equimolar amounts of both monomers into a 250 mL round bottom flask, along with a magnetic stirrer. To the flask was subsequently added 0.5 mol% of AIBN initiator with regards to the total amount of monomer and 0.5 w% of dodecanethiol with regards to the mass of both monomers. The flask was subsequently deoxygenated and added to an oil bath pre-heated to 65°C and left to react overnight. The reaction was quenched by introducing oxygen into the system.
